# Supplementary material for: A structural model of the human serotonin transporter in an outward-occluded state
Source: PLoS One. 2019 Jun 28;14(6):e0217377. doi: 10.1371/journal.pone.0217377 (PMC6599148; doi:10.1371/journal.pone.0217377)
Supplement: S1 File — (PDF) [file pone.0217377.s001.pdf]

```
>P1;hSERT_ceo
sequence:hSERT_ceo:74:A:615:A:human serotonin transporter:::
GSQGERETWGKKVDFLLSVIGYAVDLGNVWRFPYICAQNGGGAFLLPYTIMAIFGGI---------------
--------------------PLFYMELALGQYHRNGCISIWRKICPIFKGIGYAICIIAFYIASYYNTIMAW
ALYYLISSFTDQLPWTSCKNSWNTGNCTNYFSEDNITWTLHSTSPAEEFYTRHVLQIHRSKGLQDLGGISWQ
LALCIMLIFTVIYFSIWKGVKTSGKVVWVTATFPYIALSVLLVRGATLPGAWRGVLFYLKPNWQKLLETGVW
IDAAAQIFFSL---------------------------------GPGFGVLLAFASYNKFNNNCYQDALVTS
VVNCMTSFVSGFVIFTVLGYMAEMRNEDVSEVAKDA----------------------------GPSLLFIT
YAEAIANMPASTFFAIIFFLMLITLGLDSSFAGLEGVITAVLDEFPHVWAKRRERFVLAVVITCFFGSLVTL
TFGGAYVVKLLEEYATGPAVLTVALIEAVAVSWFYGITQFCRDVKEMLGFSPGWFWRICWVAISPLFLLFII
ASFLMSPPQLRLFQYNYPYWSIILGYAIGTSSFICIPTYIAYRLIITPGTFKERIIKSITPE*

>P1;hSERT_fitted_parts
structure:hSERT_fitted_parts:96:A::::::
----------------------AVDLGNVWRFPYICAQNGGGAFLLPYTIMAIFGGI/---------------
------------------------------------------------------------------------
------------------------------------------------------------------------
-------------------------------------------------AWRGVLFYLKPNWQKLLETGVW
IDAAAQIFFSL/-------------------------------------------------------------
-------VSGFVIFTVLGYMAEMRNEDVSEVAKDA/*
>P1;hSERT_5I71
structure:hSERT_5I71:74:A:615:A:human serotonin transporter:PDB: 3.15:
0.42
GSQGERETWGKKVDFLLSVIGY-----------------------------------AVDLGNVWRFPYICA
QNGGGAFLLPYTIMAIFGGIPLFYMELALGQYHRNGCISIWRKICPIFKGIGYAICIIAFYIASYYNTIMAW
ALYYLISSFTDQLPWTSCKNSWNTGNCTNYFSEDNITWTLHSTSPAEEFYTRHVLQIHRSKGLQDLGGISWQ
LALCIMLIFTVIYFSIWKGVKTSGKVVWVTATFPYIALSVLLVRGATLPG----------------------
-----------AWRGVLFYLKPNWQKLLETGVWIDAAAQIFFSLGPGFGVLLAFASYNKFNNNCYQDALVTS
VVNCMTSF----------------------------VSGFVIFTVLGYMAEMRNEDVSEVAKDAGPSLLFIT
YAEAIANMPASTFFAIIFFLMLITLGLDSSFAGLEGVITAVLDEFPHVWAKRRERFVLAVVITCFFGSLVTL
TFGGAYVVKLLEEYATGPAVLTVALIEAVAVSWFYGITQFCRDVKEMLGFSPGWFWRICWVAISPLFLLFII
ASFLMSPPQLRLFQYNYPYWSIILGYAIGTSSFICIPTYIAYRLIITPGTFKERIIKSITPE*
```
