# Supplementary material for: A structural model of the human serotonin transporter in an outward-occluded state
Source: PLoS One. 2019 Jun 28;14(6):e0217377. doi: 10.1371/journal.pone.0217377 (PMC6599148; doi:10.1371/journal.pone.0217377)
Supplement: S2 File — (PDF) [file pone.0217377.s002.pdf]

```
* Topologies generated by
* CHARMM General Force Field (CGenFF) program version 1.0.0
*
36 1

! "penalty" is the highest penalty score of the associated parameters.
! Penalties lower than 10 indicate the analogy is fair; penalties between
10
! and 50 mean some basic validation is recommended; penalties higher than
! 50 indicate poor analogy and mandate extensive validation/optimization.

RESI UNK            0.000
GROUP             ! CHARGE   CH_PENALTY
ATOM O      OG311  -0.530 !    0.075
ATOM N      NG2R51 -0.521 !    0.000
ATOM N1     NG3P3  -0.301 !   17.712
ATOM C      CG2R51 -0.048 !   11.172
ATOM C1     CG2RC0  0.156 !    5.598
ATOM C2     CG321  -0.139 !   43.839
ATOM C3     CG2RC0  0.235 !    0.000
ATOM C4     CG2R51 -0.156 !    5.566
ATOM C5     CG324   0.080 !   43.615
ATOM C6     CG2R61 -0.304 !    0.598
ATOM C7     CG2R61 -0.279 !    0.000
ATOM C8     CG2R61  0.064 !    0.075
ATOM C9     CG2R61 -0.198 !    0.000
ATOM H      HGA2    0.090 !    0.000
ATOM H1     HGA2    0.090 !    0.000
ATOM H2     HGR52   0.220 !    0.000
ATOM H3     HGP1    0.363 !    0.000
ATOM H4     HGA2    0.090 !    2.455
ATOM H5     HGA2    0.090 !    2.455
ATOM H6     HGR61   0.199 !    0.000
ATOM H7     HGR61   0.193 !    0.000
ATOM H8     HGR61   0.196 !    0.000
ATOM H9     HGP2    0.330 !    0.000
ATOM H10    HGP2    0.330 !    0.000
ATOM H11    HGP1    0.420 !    0.000
ATOM H12    HGP2    0.330 !    0.000
```

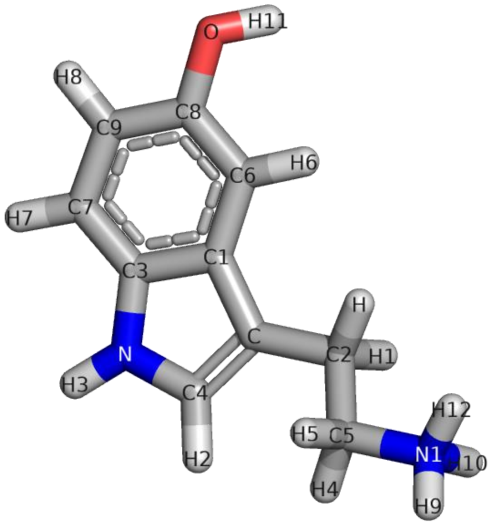


```
BOND O    C8
BOND O    H11
BOND N    C3
BOND N    C4
BOND N    H3
BOND N1   C5
BOND N1   H9
BOND N1   H10
BOND N1   H12
BOND C    C1
BOND C    C2
BOND C    C4
BOND C1   C3
BOND C1   C6
BOND C2   C5
BOND C2   H
BOND C2   H1
BOND C3   C7
BOND C4   H2
BOND C5   H4
```

```
BOND C5   H5
BOND C6   C8
BOND C6   H6
BOND C7   C9
BOND C7   H7
BOND C8   C9
BOND C9   H8

END



* Parameters generated by analogy by
* CHARMM General Force Field (CGenFF) program version 1.0.0
*

! Penalties lower than 10 indicate the analogy is fair; penalties between
10
! and 50 mean some basic validation is recommended; penalties higher than
! 50 indicate poor analogy and mandate extensive validation/optimization.

BONDS

ANGLES
CG2R51 CG321  CG324    58.35    114.00 ! m0120.Z , from CG2R51 CG321
CG314, penalty= 0.6

DIHEDRALS
CG2R51 CG2R51 CG321  CG324      0.2000  1     0.00 ! m0120.Z , from
CG2R51 CG2R51 CG321 CG314, penalty= 0.6
CG2R51 CG2R51 CG321  CG324      0.2700  2     0.00 ! m0120.Z , from
CG2R51 CG2R51 CG321 CG314, penalty= 0.6
CG2R51 CG2R51 CG321  CG324      0.0000  3     0.00 ! m0120.Z , from
CG2R51 CG2R51 CG321 CG314, penalty= 0.6
CG2RC0 CG2R51 CG321  CG324      0.0900  2   180.00 ! m0120.Z , from
CG2RC0 CG2R51 CG321 CG314, penalty= 0.6
CG2RC0 CG2R51 CG321  CG324      0.5700  3     0.00 ! m0120.Z , from
CG2RC0 CG2R51 CG321 CG314, penalty= 0.6
CG2RC0 CG2R61 CG2R61 OG311      3.1000  2   180.00 ! m0120.Z , from
CG2R61 CG2R61 CG2R61 OG311, penalty= 1.5
CG2R51 CG321  CG324  NG3P3      0.2000  3     0.00 ! m0120.Z , from NG3P3
CG314 CG321 CG2R51, penalty= 4
CG2R51 CG321  CG324  HGA2       0.2000  3     0.00 ! m0120.Z , from HGA1
CG314 CG321 CG2R51, penalty= 4.1

IMPROPERS

END
```
