# Supplementary material for: A structural model of the human serotonin transporter in an outward-occluded state
Source: PLoS One. 2019 Jun 28;14(6):e0217377. doi: 10.1371/journal.pone.0217377 (PMC6599148; doi:10.1371/journal.pone.0217377)
Supplement: S1 Table — (PDF) [file pone.0217377.s003.pdf]

S1 Table. Similarity of structural elements in hSERT and LeuT

	Residue range (hSERT)	Residue range (LeuT)	hSERT to LeuT before fitting	hSERT to LeuT after fitting	hSERT to hSERT after fitting	Sequence identity (%)
Hash domain	157-191, 253-273, 420-455, 462-481	88-122, 165-185, 337-372, 375-394	-	2.8	-	-
TM1b2top	96-130	22-56	3.9	0.7	3.8	57.1
EL3TM6a	305-313, 314-321, 322-337	223-231, 232-239, 240-255	2.9	0.4	2.9	48.5
TM7topEL4a	374-401	292-319	5.3	3.5	4.1	14.3
Upper bundle (all)			4.1	1.9	3.6	41.7

Results are reported as RMSDs (Å) and sequence identity (%) of the aligned segments. Note that the EL3TM6a segment was divided into three parts for the structure alignment.
