# Supplementary material for: A structural model of the human serotonin transporter in an outward-occluded state
Source: PLoS One. 2019 Jun 28;14(6):e0217377. doi: 10.1371/journal.pone.0217377 (PMC6599148; doi:10.1371/journal.pone.0217377)
Supplement: S2 Table — (PDF) [file pone.0217377.s004.pdf]

S2 Table. Ligand-protein distance restraints used for equilibration

Atom pair	Distance (Å)	Atom pair	Distance (Å)
Na1 – A96/O	2.3	Cl – S372/OG	3.0
Na1 – N101/OD1	2.6	HOH901/O – Na2	2.5
Na1 – S336/O	2.5	HOH901/O – D437/OD2	2.8
Na2 – G94/O	2.5	5HT/N1 – D98/OD2	2.8
Na2 – V97/O	2.3	5HT/O – T439/OG1	2.8
Na2 – L434/O	2.5	Y176/CG – F335/CG	8.0
Cl – Y121/OH	3.0	R104/NH2 – E493/OE1	3.0
Cl – Q332/OG	3.5	R104/NH1 – E493/OE2	2.6

Distances were taken from known structures of hSERT and applied as restraints between the listed atoms.
