# Supplementary material for: A structural model of the human serotonin transporter in an outward-occluded state
Source: PLoS One. 2019 Jun 28;14(6):e0217377. doi: 10.1371/journal.pone.0217377 (PMC6599148; doi:10.1371/journal.pone.0217377)
Supplement: S3 Table — (PDF) [file pone.0217377.s005.pdf]

S3 Table. Structure quality scores and ion-protein relevant distances

Table A – Structure quality scores and ion-protein distances for the hSERT outward-occluded models after energy minimization

Model number	Clash score	MolProbity score (Å)	Allow (%)	Na1 – N368/OD1 dist (Å)	Cl – S336/HG1 dist (Å)	Cl – S372/HG1 dist (Å)	Cl – Y121/HH dist (Å)	F335 rotamer
0120*	2.52	1.69	95.43	2.8	2.6	2.7	2.5	
1354*	2.52	1.60	96.19	2.7	2.6	2.7	2.4	
1362*	3.12	1.75	95.05	2.8	2.6	2.5	2.4	
1473	2.88	1.74	95.24	2.8	2.4	2.7	**3.8**	**flipped**
1726	2.88	1.61	95.62	**3.3**	2.4	**3.8**	2.4	
1748	2.16	1.52	95.62	**3.3**	2.8	2.6	**3.8**	**flipped**
1768	2.40	1.63	95.62	2.8	2.5	**4.4**	3.5	
1833	2.52	1.67	95.62	2.7	**3.8**	2.6	**3.7**	

*The three selected models. Entries in bold indicate the reason for exclusion of a given model. Allow = percentage of residues whose backbone dihedrals are in the allowed regions of the Ramachandran plot.

Table B – Structure quality scores for hSERT models prior to energy minimization

Model or structure	Clash score	MolProbity score (Å)	Allow (%)
0120	80.29	2.55	96.95
1354	79.09	2.57	96.76
1362	75.01	2.68	97.14
1473	74.05	2.56	96.57
1726	76.21	2.51	97.14
1748	77.05	2.45	97.52
1768	74.65	2.60	96.19
1833	73.21	2.56	96.57
5I71*	31.17	2.23	93.25
5I71-min	5.51	1.72	96.38

*5HT-bound hSERT outward-open structure (5I71) before energy minimization; 5I71-min = after energy minimization.
