# Supplementary material for: A structural model of the human serotonin transporter in an outward-occluded state
Source: PLoS One. 2019 Jun 28;14(6):e0217377. doi: 10.1371/journal.pone.0217377 (PMC6599148; doi:10.1371/journal.pone.0217377)
Supplement: S4 Table — (PDF) [file pone.0217377.s006.pdf]

S4 Table. Docking and clustering results for the hSERT outward-occluded model

Table A – All induced-fit docking poses

Model number	Number of poses	Glide Gscore (kcal/mol)	IFD score (kcal/mol)
120	73	–8.2 ± 0.5	–16629 ± 610
1362	65	–7.9 ± 0.6	–16589 ± 713

Total number of poses for each of the outward-facing models, as well as the mean ± standard deviations of the Glide Gscore and IFD scores for those poses.

Table B – Most-populated clusters from induced-fit docking

Cluster number	Number of poses	Glide Gscore (kcal/mol)	IFD score (kcal/mol)	Models present (pose number per model)
1	21	–8.0 ± 0.7	–15557 ± 16	1362 (21)
8	57	–8.4 ± 0.5	–17081 ± 30	120 (32); 1362 (25)
9	22	–8.1 ± 0.4	–15701 ± 17	120 (22)

The most-populated clusters are defined as those containing >15% of all poses (i.e. >20 poses per cluster). The number of poses per cluster, and the number of poses per model in that cluster, are reported alongside the mean ± standard deviations of the Glide Gscore and IFD scores for all poses in that cluster.
