# Supplementary material for: A structural model of the human serotonin transporter in an outward-occluded state
Source: PLoS One. 2019 Jun 28;14(6):e0217377. doi: 10.1371/journal.pone.0217377 (PMC6599148; doi:10.1371/journal.pone.0217377)
Supplement: S5 Table — (PDF) [file pone.0217377.s007.pdf]

S5 Table. Docking and clustering data for the outward-open structure (5I71)

Cluster	Number of poses	Glide Gscore (kcal/mol)	IFD score (kcal/mol)
All	74	–7.8 (0.6)	–18169 (16)
7	30	–8.4 (0.3)	–18182 (11)
8	11	–7.9 (0.4)	–18168 (15)

The most-populated clusters are defined as those containing >15% of all poses (i.e. >10 poses per cluster). The number of poses per cluster (or for the whole dataset) are reported alongside the mean ± standard deviations of the Glide Gscore and IFD scores for all poses in that cluster.
