# Supplementary figures and images for: A structural model of the human serotonin transporter in an outward-occluded state
Source: PLoS One. 2019 Jun 28;14(6):e0217377. doi: 10.1371/journal.pone.0217377 (PMC6599148; doi:10.1371/journal.pone.0217377)

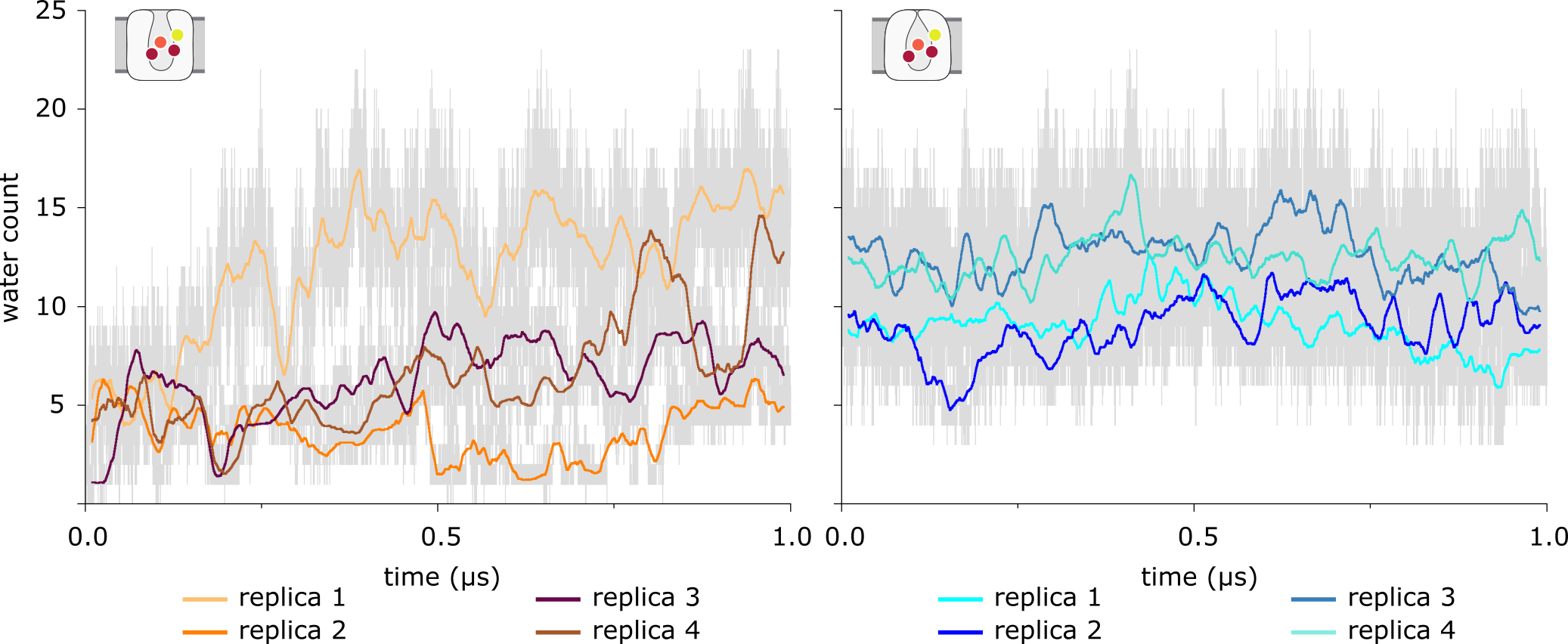

Supplement: S1 Fig — Number of waters in the intracellular vestibule during molecular dynamics simulations of hSERT, as a function of time. The plots show the underlying data for the outward-open (left) and outward-occluded (right) trajectories used to plot the distribution of the intracellular water counts in Fig 10A of the main manuscript. (TIF) [file pone.0217377.s008.tif]

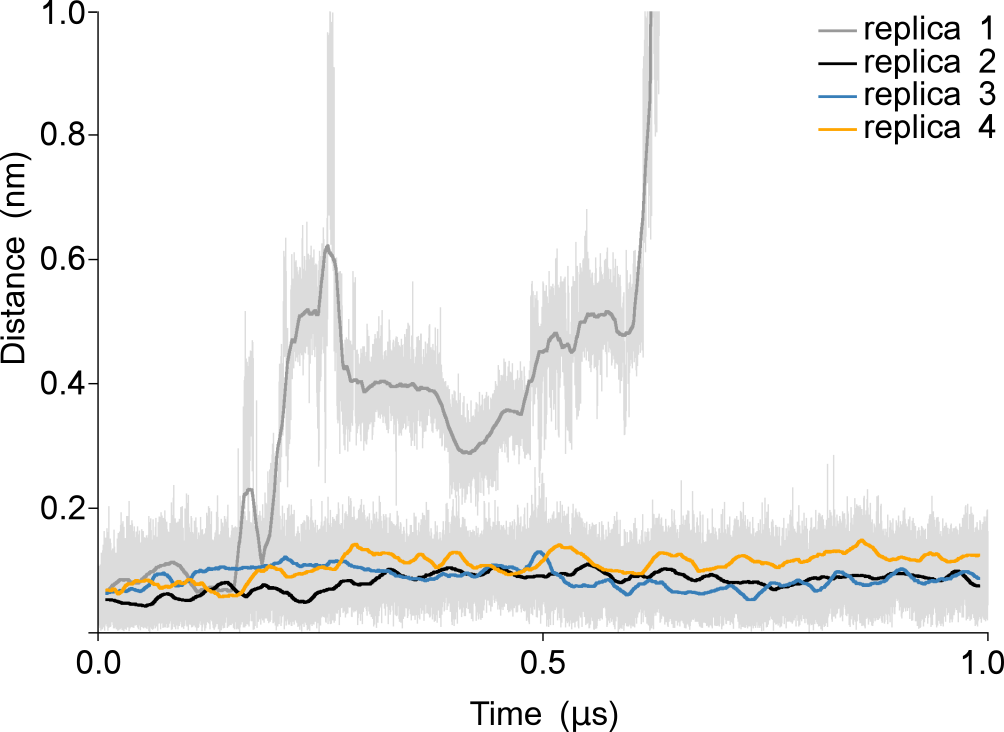

Supplement: S2 Fig — Distance of the chloride ion from its initial position during the simulations of the outward-open model. The protein was least squares fitted to the initial frame of each of four replicas. (TIF) [file pone.0217377.s009.tif]
